# Seroprevalence of Hepatitis B Infection Among Blood Donors in a Tribal-Preponderant State of India: A Seven-Year Retrospective Observational Study

**DOI:** 10.7759/cureus.66404

**Published:** 2024-08-07

**Authors:** Sushma Kumari, Abhay Kumar, Siddharth Kapoor, Usha Saroj, Saket Verma, Divakar Kumar

**Affiliations:** 1 Blood Bank, Rajendra Institute of Medical Sciences, Ranchi, IND; 2 General Medicine, Rajendra Institute of Medical Sciences, Ranchi, IND; 3 Biochemistry (Trauma Centre), Rajendra Institute of Medical Sciences, Ranchi, IND; 4 Internal Medicine, Rajendra Institute of Medical Sciences, Ranchi, IND

**Keywords:** viral hepatitis, hepatitis, hbv, tti, blood safety, transfusion transmitted infection, hepatitis b infection

## Abstract

Background

Blood transfusion is an essential and lifesaving procedure for many acute and chronic diseases. Though saving millions of lives, it carries the risk of transfusion of transfusion-transmitted infections (TTIs), including hepatitis B. Detection of this infection prior to transfusion saves potentially vulnerable patients from an additional burden and prevents the further spread of disease.

Aim and objectives

Our present study aimed to determine the seroprevalence of hepatitis B virus (HBV) in blood donors at the Rajendra Institute of Medical Sciences (RIMS) in Jharkhand, a tribal-preponderant state of India.

Materials and methods

After obtaining approval from the institutional ethics committee, a retrospective observational study was conducted among the eligible blood donors visiting RIMS from April 2016 to March 2023. A total of 195,507 subjects were included in the study. All blood donation samples collected in ethylenediaminetetraacetic acid (EDTA) vials were tested for five TTIs: human immunodeficiency virus 1 and 2, HBV, hepatitis C virus (HCV), malaria and syphilis. HBV testing was conducted via chemiluminescence technique to check f or the presence of hepatitis B surface antigen (HBsAg) in plasma.

Results

Among the study sample of 195,507 donors, the prevalence of HBsAg positivity was 0.87%. Among all the TTIs, more than 50% (51.93%) were HBsAg positive. The positivity percentage was higher in male donors and HBsAg positivity rose with an increase in replacement donors.

Conclusions

HBV is a major health concern in developing countries such as India due to its high endemicity. Therefore, early detection of HBV carriers in the blood donor population helps in curbing the spread of further infection and it also helps policymakers to develop different health programs to reduce further incidence of the infection in the general population.

## Introduction

Blood transfusions are lifesaving for many chronically ill patients and save millions of lives every year. They are also beneficial in the treatment of transfusion-dependent thalassemia, sickle cell anaemia, chronic dialysis patients, etc. However, transfusions come with the risk of mild-to-severe life-threatening complications due to transfusion-transmitted infections (TTIs). To prevent the transmission of infection via blood transfusion, the World Health Organization makes HIV 1 and 2, hepatitis B, hepatitis C, malaria, and syphilis testing mandatory for all donors. Testing of these infections along with screening of blood donors also provides data on disease prevalence [1].

Hepatitis B virus (HBV) is a 42 nm Hepadna virus transmitted by infected blood and blood products. HBV is present in several other body fluids, such as semen and vaginal secretions, which causes its transmission via sexual contact. Perinatal transmission is very common in areas with high endemicity [2,3]. The prevalence is high in male-to-male (MSM) sex workers, intravenous drug users, and healthcare workers who are prone to needle pricks or handling blood or other body fluids [2].

Infection with HBV causes both acute and chronic hepatitis. Liver failure can occur as a sequel to severe acute hepatitis and may be fatal in many cases. Most patients suffering from acute hepatitis recover, while in some it continues as chronic hepatitis. In its chronic phase, it can progress to liver cirrhosis and hepatocellular carcinoma [4].

Our present study was done in the blood centre of Jharkhand, a tribal-preponderant state of India with 32 tribes. As per the 2011 census, the tribal population comprises 26.3% of the total population of the state, and most of them reside in villages. Around 41.6% of the tribal population reside in Ranchi and Pakur districts [5]. This study was done to see the prevalence of HBV over a long period of time in blood donors, which also reflects its prevalence in the general population of the region. Our study will help policymakers in formulating policies and implementing health programs regarding the implementation of preventive measures to reduce the incidence of HBV by preventing further spread in the population.

## Materials and methods

Study settings and population

A seven-year retrospective observational study was conducted among the blood donors visiting the blood bank of Rajendra Institute of Medical Sciences (RIMS), a tertiary health care centre and teaching hospital serving the state of Jharkhand and its neighbouring tribal-preponderant states. After obtaining approval from the institutional ethics committee of RIMS, the data were searched from the blood centre records, both from the master register and the discard register. The period of study was from April 2016 to March 2023.

Sample collection and processing

Clinically healthy blood donors of 18-65 years of age weighing more than 45 kg and with a haemoglobin level ≥ 12.5 gm%, who gave consent and fulfilled donor selection criteria of National Blood Transfusion Council (NBTC) guidelines were included in the study. Donors who did not fulfil the donor selection criteria, such as having jaundice, history of chronic hepatic, renal, or cardiac ailments, history of epilepsy, allergy, or any high-risk behaviour such as intravenous drug use and having multiple sex partners as per NBTC guidelines were not allowed to donate blood and were excluded from the study [6]. In our study, voluntary donors were categorized as those who donate without expecting any payment or rewards in return, mostly donating in blood donation camps, while replacement donors are those who donate blood when it is required for a patient. Blood samples were collected in EDTA vials after blood donation from all the eligible donors, who were selected after proper history taking and a physical examination by a medical officer.

Screening of samples and method of HBV detection

Every donor’s sample was tested for the five TTIs, including HBV. Screening of HBV was done by using the third-generation kit of Abbott diagnostics by chemiluminescence technique (Model-Architect i1000SR, Abbott Laboratories, Chicago, IL), which tests for HBsAg antigen present in plasma by using chemiluminescent microparticle immunoassay (CMIA) and gives results in relative light units (RLU). The amount of HBsAg antigen present in serum or plasma was reflected in RLU. During the testing of samples, the manufacturer’s instructions were strictly followed. Samples showing values > 1RLU were considered positive for HBsAg antigen. Values between 0.8 and 0.99 were considered to be in the grey zone and retested. Blood units that tested positive and were in the grey zone were discarded, and donors who were found positive were counselled and asked to consult a physician for further evaluation. Those donors whose results were found in the grey zone even after repeat testing were counselled and asked to repeat the test with the nucleic acid technique.

Data collection and statistical analysis

Data from April 2016 to March 2023 regarding age, sex, type of donor, month and year of donation and HBsAg positivity were obtained from data stored in the records of the blood centre. After reviewing the records of 195,507 donors, the data were extracted and studied.

Data was entered in Microsoft Excel, and data analysis was performed using SPSS v. 22.0 (IBM Corp., Armonk, NY). Tables were prepared from available data. A chi-square test was used for analytical assessment, and a P-value ≤ 0.01 was considered statistically significant. The Wilson score interval test was used to estimate the proportion with a 95% confidence interval (95% CI).

Ethical approval

Ethical approval was provided by the institutional ethics committee via letter number 158 of the Rajendra Institute of Medical Sciences dated May 10, 2024.

## Results

Our study data of seven years from April 2016 to March 2023 included a total of 195,507 blood units collected from both voluntary and replacement donors. Among these 146,478 were replacement donors and 49,489 were voluntary donors. Male donors (179,313) were predominant over female donors (16,194) throughout the study period (Table 1).

**Table 1:** Distribution of blood donors according to year, type, and sex The chi-square static is 2073.0267 and the p-value is <0.00001. Data were shown as n and n(%). A p-value of <.05% was considered significant. VD = voluntary donors, RD = replacement donors; n = numbers; % = percentage

Year	Total blood units collected both from VDs and RDs (n)	Number of blood units collected from VDs (n)	Number of blood units collected from RDs (n)	Number of blood units collected from male donors (n)	Number of blood units collected from female donors (n)
April 2016 - March 2017	27,960	8,615	19,345	25,330	2,630
April 2017 - March 2018	28,374	9,053	19,321	25,494	2,880
April 2018 - March 2019	30,226	9,962	20,264	26,829	3,397
April 2019 - March 2020	32,596	8,683	23,913	29,314	3,282
April 2020 - March 2021	20,579	3,642	16,937	19,209	1,370
April 2021 - March 2022	24,892	4,603	21,289	23,544	1,348
April 2022 - March 2023	30,880	4,931	25,949	29,593	1,287
Total, n(%)	195,507 (100%)	49,489 (24.81%)	14,7018 (75.19%)	179,313 (91.71%)	16,194 (8.29%)

An increasing trend in blood donation was seen from April 2016 to March 2019 and sharply declined in April 2020. After March 2021, a rise in the blood donation pattern was seen. The highest number of voluntary donations was seen from March 2018 to April 2019 (32.96%), which shows a declining trend in the coming years except during the April 2021 to March 2022, period where a mild increase in VD was seen. Replacement donations showed an increasing trend in chronicity (Table 1).

Male donors (91.72%) were more than female donors throughout the study period (8.2%). Participation of female donors increases progressively until March 2019 (11.24%); after this period, a declining trend can be seen until 2023 (4.17%) (Table 1).

Among a total of 195,507 donors, 3273 (1.67%) donors tested positive for the following infectious diseases: HIV, HBV, hepatitis C, malaria, and syphilis (Table 2).

**Table 2:** Year-wise prevalence of total TTIs and HBV among blood donors Data are presented as numbers (n) and proportions with a 95% confidence interval (CI); proportions are represented as percentages. The Wilson score interval test is used to estimate the proportion with 95% CI. TTI: transfusion-transmitted infection, CI: confidence interval, HBV: hepatitis B virus

Year range	Total blood units collected (n)	Total TTIs (n)	Proportion of TTIs (95%CI)	Total HBV blood donors (n)	Proportion of HBV positivity (95%CI)
April 2016 - March 2017	27,960	490	1.75 (1.60-1.91)	256	0.92(0.81-1.08)
April 2017 - March 2018	28,374	487	1.72(1.57-1.87	263	0.93(0.82-1.05)
April 2018 - March 2019	30,226	464	1.54(1.40-1.68	255	0.84(0.74-0.95)
April 2019 - March 2020	32,596	490	1.50(1.37-1.64)	251	0.77(0.68- 0.88)
April 2020 - March 21	20,579	393	1.91(1.73-2.11)	154	0.75(0.64-0.88)
April 2021 - March 2022	24,892	447	1.80(1.63-1.97)	247	0.99(0.87-0.12)
April 2022 - March 2023	30,880	502	1.63(1.49-1.77)	275	0.89(0.79-1.00)
Total	195,507	3273	1.67(1.62-1.73)	1701	0.87(0.83-0.91)

Among these, 1,701 donors tested positive for HBsAg, a marker of HBV, which is 0.87% of total blood donors and 51.97% of total TTI-positive donors. The maximum percentage of TTI positivity was seen from April 2020 to March 2021 (1.91%), and the minimum number was from April 2019 to March 2020. The data shows a decreasing trend in TTIs from April 2016 to March 2020. Then a sharp rise in TTIs was seen from April 2020 to March 2021, and again a declining trend was seen until March 2023. A declining trend of HBsAg positivity was seen among blood donors from the inception of the study period till March 2021. Thereafter, a sharp increase in HBV-positive donors was seen from 0.75% to 0.99% between April 2021 and March 2022. However, the total number of TTI-positive donors showed a declining trend until March 2020 but here a rise in positive donors is seen from April 2019 to March 2020. In 2017 and 2018, a declining trend is seen in both total TTI-positive and HBV-positive donors. From April 2020 to March 2021, there was the highest number of TTI positivity but the lowest proportion of HBV positivity among blood donors. From April 2021 to March 2022, there was a sharp decline in voluntary blood donation and a rise in HBV positivity, which reached a peak of 0.99%. It again rose in 2021, showing its highest HBsAg positivity at 0.97%. Again there was a declining trend from April 2022 to 2023 in both total TTI-positive cases as well as HBV-positive cases (1.63% and 0.89 % respectively). Among a total of 4,161 TTI-positive donors, all were male donors; this was seen among HBV-positive donors (Table 3).

**Table 3:** Year and gender-wise distribution of hepatitis B-positive blood donors Data are presented as numbers(n) and proportion with 95% CI; proportion is represented as percentage. The Wilson score test was used to estimate the proportion with 95% CI. TTI: transfusion-transmitted infection, CI: confidence interval, HBV: hepatitis B virus

Year	Total TTI-positive donors (n)	Total HBV-positive male donors (n)	Proportion of HBV-positive male donors with 95% CI	Total HBV-positive female donors (n)	Proportion of HBV-positive female donors with 95% CI
April 2016 -March 2017	490	256	52.44(47.72-56.74)	0	0
April 2017 -March 2018	487	263	54.00(49.46-58.50)	0	0
April 2018 - March 2019	464	255	54.96(50.30-59.55)	0	0
April 2019 -March 2020	490	251	51.22(46.70-55.73)	0	0
April 2020 - March 2021	393	154	39.19(34.33-44.21)	0	0
April 2021 - March 22	447	247	0.55.26(50.51-59.93)	0	0
April 2022 - March 2023	502	275	54.78(50.31-59.20)	0	0
Total	3,273	1,701	51.97(50.24-53.70)	0	0

## Discussion

Globally, HBV is a major health-related issue. The most commonly affected WHO regions in decreasing order of prevalence are the Western Pacific region (97 million), African region (65 million), South East Asian region (61 million), Eastern Mediterranean region (15 million), European region (11 million), and America (95 million). According to the data of the Global Hepatitis Report, about 254 million people are infected with HBV and data from 187 WHO countries depicts that mortality from viral hepatitis infection rose from 1.1 million in 2019 to 1.3 million in 2022. HBV is accountable for 83% of fatal cases. New cases are coming mostly from African countries (63%), among which new cases were seen mostly in people of the 30- to 54-year-old age group is the main blood donor pool [4].

On the basis of the prevalence of HBV countries are categorized into different zones of prevalence, such as low (<2%), intermediate (2-7%) and high (≥8%) endemicity zones. India, with a 4% prevalence, lies in the intermediate zone. India, which accounts for about one-fifth of the world's population, carries a wider burden of HBV by sheltering 40 million HBV carriers, which is about 10-15% of total HBV carriers worldwide [7]. Early detection of these carriers will help in lowering the burden of HBV infection, its dreaded complications, and further associated mortality.

In our study, replacement donation is the main form of donation (75.2%). Similar data on the high prevalence of male donors was shown by studies done in different parts of India. The highest prevalence was seen in the study done by Pahuja et al. in Delhi (99.48%)[8], followed by Garg et al. in Rajasthan(90%) [9], Pawan et al. in New Delhi (84.87%)[10], Singh et al. in coastal Karnataka (84.43%)[11], Pokhrel et al. in north India (80.3%) [12], Sonam K. from Punjab (71.5%)[13], and Arora et al. in Haryana (68.6%) [14]. However, contrasting data showing a higher proportion of voluntary donations can be seen in several studies. In a study done by Patel et al. in Gujarat, 98.56% [15] of donors are voluntary donors, 80.4% voluntary donors are seen in a study from Rajasthan by Dhariwal et al., 69.6% in a study from Jammu and Kashmir, 65.86% from Punjab [16-18], and 68.7% from Sundaramoorthy et al. in Andhra Pradesh [19].

Despite the initial increase in the percentage of voluntary donation, indicating a rise in blood donation awareness among the general population, it is often less than RD. During the COVID-19 pandemic (2020-2021), there was a decline in voluntary donations. The main factor behind this is a fear in people of getting infected and there was also a decrease in voluntary blood donation camps, which were the main source of voluntary donors. Later, voluntary donations increased due to an increase in voluntary blood donation camps, but still far below the pre-COVID period.

In our study, male donors (91.7%) are more than female donors (8.3%). Similar data was shown in studies done by Boubker et al. in Morocco (72%), and Sumbu et al. (67.7%) [20,21]. The percentage of female donors grew steadily till 2019 but reduced drastically in 2020 during the COVID-19 pandemic period and went on to decline thereafter. The same trend is seen in the study done by Thakur et al. (99.12%) [22], Mondal et al. (98.3%) [23], Kaur et al. (98.17%) [18], Ryhan (98.45%) [17], Sonam et al. (96.8%) [13], Pawan et al. (96.12) [10], Dhariwal et al. (97.5%) [16], Pahuja et al. (97.24%) [8], Saini et al. (99.7%) [24], and Shah et al. (99.08%) [25]. 

Women who come to our tertiary centre are hesitant to donate blood citing fears of experiencing weakness and being unable to fulfil their household duties. However, there seems to be a shift in perception as our results show an increasing trend of participation by females. Females who come forward to donate blood in our centre are usually deferred due to lower haemoglobin concentration and lower body weight. Other deferral criteria prevailing in female donors in our centre are menstruation, pregnancy, abortion, lactation etc.

In the present study, the prevalence of TTI is 1.66%, while that of HBV is 0.85%. In our study, HBV emerged as the most common infection among all TTIs; more than 50% of TTI-positive units belonged to HBV-positive donors (51.33%). The prevalence rose progressively from April 2016 to March 2018 but then showed a declining trend until 2020 during the COVID-19 pandemic period. Subsequently, it showed an increasing trend after April 2021 due to a decrease in COVID-19 cases, then a mild decrease was seen in 2023 which is not very significant. The same increasing trend of HBV was seen in the study done by Kumari et al. [13] with 39.5% positivity among all TTIs. However, the prevalence of HBV is less than in our study and is not the most common TTI as seen in ours. In a study done in Maharashtra by Gurwale et al., the prevalence of HBsAg-positive donors was 0.62%. The HBsAg positivity rate was higher among the males (0.7%) [26]. Another study by Jagannathan et al. from Bangalore shows 0.94% HBsAg positivity among blood donors [27]. Another study by Giriyan and Sindhushree from Karnataka shows a 2% prevalence [28] but here also more positivity is seen among male donors. A study done by Bhaumik showed data on HBsAg positivity among blood donors from different studies done in different regions of India, ranging from 0.2 to 2.9 from 2004 to 2014 [29]. In a 2007 meta-analysis by Batham et al., the prevalence of HBV among the tribal population was 15.9% in comparison to 2.4% in the nontribal population [30].

In our study, TTI and HBsAg positivity were higher in males than females and were statistically significant. Similar data was seen in most of the studies done earlier, but contrast is seen in the study done by Sundaramoorthy et al. where TTI positivity was more in females [19]. The reason for the higher prevalence of HBV positivity in male donors could be due to the higher ratio of male-to-female donors in the entire donor population throughout the study. Less positivity in females could be due to HBV vaccination in females during pregnancy.

To prevent TTIs and increase blood safety, it is essential to increase voluntary blood donation. In our study, HBsAg positivity was in parallel with replacement donation, showing the need to increase voluntary blood donation to prevent HBV infection.

HBV and other TTIs that are prevalent in society can be curtailed by increasing awareness among the general population regarding voluntary blood donation, regular and rigorous pre-donation counselling, and maintaining confidentiality during counselling so that donors can readily disclose histories of high-risk behaviours such as having multiple sex partners or needle-sharing. Shifting to more sensitive tests, such as nucleic acid tests (NAT) for early detection of the viral genome and strict monitoring of TTI-positive cases, is vital in curtailing the disease spread and ensuring proper treatment.

Limitations

For the detection of HBV, only HBsAg antigen-detected cases were taken as positive. Carriers in the window period were missed. Genetic variant study was also not done.

## Conclusions

An increase in awareness regarding voluntary blood donation and the use of more specific tests such as the NAT test is required to increase blood safety. Early detection of HBV carriers in countries that lie in intermediate endemicity zones will help curtail the spread of infection in the community and help policymakers in the development, allocation, proper implementation and resource utilization of different health programs in tribal regions for the prevention and treatment of HBV.
